# *‘Researchers have love for life’*: opportunities and barriers to engage pregnant women in malaria research in post-Ebola Liberia

**DOI:** 10.1186/s12936-018-2292-7

**Published:** 2018-04-02

**Authors:** Guillermo Martínez Pérez, Christine K. Tarr-Attia, Bondey Breeze-Barry, Adelaida Sarukhan, Dawoh Peter Lansana, Ana Meyer García-Sípido, Anna Rosés, María Maixenchs, Quique Bassat, Alfredo Mayor

**Affiliations:** 1 ISGlobal, Hospital Clínic - Universitat de Barcelona, Barcelona, Spain; 2 Saint Joseph’s Catholic Hospital, Tubman Boulevard, Oldest Congo Town, PO Box 10-512, 1100 Monrovia, Liberia; 3 NGO Juan Ciudad Foundation, Madrid, Spain; 4 ICREA, Pg. Lluís Companys 23, 08010 Barcelona, Spain; 5 Centro de Investigação em Saúde de Manhiça (CISM), Maputo, Mozambique; 6 Pediatric Infectious Diseases Unit, Pediatrics Department, Hospital Sant Joan de Déu, Barcelona, Spain

**Keywords:** Malaria, Liberia, Qualitative research, Pregnant women, Prevention, Access to healthcare

## Abstract

**Background:**

Adoption of prevention and therapeutic innovations to ensure that National Malaria Control Programmes meet their incidence reduction targets is highly dependent on the conduct of rigorous clinical trials. In Liberia, malaria control virtually halted during the recent Ebola epidemic, and could enormously benefit from innovations to protect its most vulnerable populations, including pregnant women, against malaria. Health policy-planners could feel more inclined to adopt novel interventions with demonstrated safety and efficacy when trialled among their women population. However, pregnant women are especially vulnerable when targeted as research participants. Whilst some studies in the region attempted to understand the ethical issues around the conduct of clinical research, there is need of such information from Liberia to inform future malaria research.

**Methods:**

This is a grounded theory study that aims to understand the barriers and opportunities for pregnant women to consent to participate in malaria research in Liberia. The study was conducted between November 2016 and May 2017 at the St Joseph’s Catholic Hospital, Monrovia. In-depth interviews and focus group discussions were held with hospital staff, traditional community representatives, and pregnant women.

**Results:**

According to the participants, useful strategies to motivate pregnant women to consent to participate in malaria research could be providing evidence-based education on malaria and research to the general population and encouraging engagement of traditional leaders in research design and community mobilization. Fears and suspicions towards research and researchers, which were amplified during the conduct of Ebola vaccine and drug clinical trials, may influence women’s acceptance and willingness to engage in malaria research. Population’s mistrust in the public healthcare system might hinder their acceptance of research, undermining the probability of their benefiting from any improved malaria control intervention.

**Conclusion:**

Benchmarking for acceptable practices from previous public health interventions; building community discussion and dissemination platforms; and mapping communication and information errors from how previous research interventions were explained to the Liberian population, are strategies that might help ensure a safe and fully informed participation of pregnant women in malaria research. Inequity issues impeding access and use of biomedical care for women must be tackled urgently.

## Background

In Liberia, malaria is one of the most prevalent causes of death [1]. With a resource-constrained health system as a result of a decade-long civil war [2], Liberia needed massive foreign aid to control the recent 2014–2016 Ebola epidemics [3], which left over 4000 deaths [4] and depleted its already scarce healthcare workforce [5]. The crisis resulted in a disruption in the provision of malaria control interventions that led to an increase of malaria-attributable mortality [6, 7]. Pregnant women, a population particularly susceptible to malaria infection [1, 8, 9], suffered the closure of health facilities and the fear of qualified professionals to assist their deliveries [10]. In spite of efforts to scale up malaria prevention after the epidemic, uptake of intermittent preventive treatment in pregnancy (IPTp) and insecticide-treated nets (ITN) by pregnant women has not increased substantially. According to the 2011 and 2016 Malaria Indicator Surveys, the percentage of women who had taken two or more doses of sulfadoxine–pyrimethamine-based IPTp increased from 50.0 to 54.5% and the percentage of women who slept under an ITN the night before increased from 39 to 40% [11, 12].

Advances to protect pregnant women against malaria depend on the development of efficient vector control as well as preventive and therapeutic strategies [9]. It has been shown that IPTp has a great potential to reduce the incidence and consequences of malaria in pregnant women [13–15] and that it can prevent low birth weight [16] or premature death among their offspring [16–18]. However, as evidenced by national survey data, full coverage of IPTp is difficult to achieve. Therefore, there appears to be a need to design innovative strategies to improve malaria prevention during pregnancy [1, 8, 9]. In order to convince National Malaria Control Programme (NMCP) planners of the utility of novel interventions for pregnant women, it is important that they are trialled in-country in the frame of rigorous research. However, in addition to being especially vulnerable to malaria infection, pregnant women are also a vulnerable study population that deserves special protection measures [19–22].

During the Ebola epidemic, a number of vaccine and drug clinical trials were initiated [23–25]. There was little time for thorough planning of research due to the urgency to find biomedical solutions to halt the spread of the virus [25]. The trials signified the arrival of funds, equipment, laboratories, researchers and knowledge [26]. Whilst some trials were prematurely terminated due to the impossibility to continue recruitment of Ebola-infected subjects [24, 27], other trials gave origin to research platforms that persist today. One such platform is the ‘Liberia–US Clinical Trials Partnership Program, Partnership for Research on Ebola Vaccines in Liberia’ (PREVAIL) [23, 28]. The maintenance of research activities has given the opportunity to look back and reflect on the management of patients during the Ebola outbreak, the conduct of clinical trials, the provision of routine healthcare services to the general population, and how traditional cultural values might have affected how the Liberians perceive researchers and research activities involving community participation and data and specimen collection.

The conditions under which vulnerable populations are recruited as study participants have long been of concern to bioethicists and trialists [27, 29, 30]. A number of qualitative studies have been conducted in a variety of settings, including Burkina Faso [31], Gabon [32], Ghana [33–35], Kenya [36, 37], or The Gambia [38, 39], to understand the impact of research on the populations’ value placed in research [38]; researchers’ expressed challenges to apply ethical principles [32]; efficacy of informed consent process [31, 36]; the influence of the community leaders and household heads in individuals’ decision to consent [33, 39]; or in parental and male partners’ comprehension of the consent process [35, 37]. Another issue of growing concern is how the exclusion of pregnant women from vaccine and drug trials may foster—rather than minimize—their susceptibility to further ill health [27].

There is need for such type of qualitative studies for Liberia that analyse—beyond Ebola—the conditions under which future clinical and socio-behavioural research on other prevalent infectious diseases (e.g. malaria) will be conducted. Together with baseline epidemiological data, socio-anthropological data on how Liberian communities experience and understand research is indispensable to inform future research with the aim of enhancing malaria prevention and control, particularly among pregnant women.

## Methods

### Aim, site and period

A qualitative inquiry with the aim to understand the barriers and opportunities for pregnant women to participate in malaria research in Liberia was conducted. This article reports on community members’ perceptions and attitudes towards research as well as on the contextual aspects that may deter or motivate pregnant women to participate in malaria research.

The study was conducted between November 2016 and May 2017 in parallel with a cross-sectional study on the prevalence of malaria among pregnant women attending antenatal care at the Saint Joseph’s Catholic Hospital (SJCH). The SJCH is a not-for-profit maternal referral hospital in Congo Town neighbourhood, in Monrovia. Monrovia, Liberia’s capital, is the largest city in Montserrado County, one of the 15 administrative divisions of the country.

In mid-2016, the SJCH, with support from the Barcelona Institute for Global Health (ISGlobal), trained in medical research ethics a group of traditional community representatives and constituted a Community Advisory Board (C.A.B). The SJCH C.A.B members provided advice for the design of this qualitative study.

### Sampling

Three groups of key-informants (KI) were invited to participate: (KI1) SJCH medical, laboratory, and management staff: (KI2) Traditional community representatives participants to the C.A.B. outreach activities: (KI3) Pregnant women that previously participated in the malaria prevalence study. All those younger than 18 years and unwilling to give consent were excluded.

Convenience sampling was used to approach, face by face, informants for KI1 and KI2 groups. Purposive sampling served to approach, by phone, informants for group KI3. Study recruitment forms from the malaria prevalence study were used to generate a randomized list of pregnant women to approach in this study.

The purpose of the study was explained and, if interested, a date was scheduled for the approached individual to meet the social scientist (GMP) at the SJCH facilities. At the scheduled date, informed consent was obtained. Two different information sheet and consent forms were used: one for the in-depth interviews (IDIs) and one for the focus group discussions (FGDs). Participants to the FGDs were reminded of the importance of keeping information shared by the other participants confidential. During the consent process, all informants were made aware of their rights to withdraw from the study at any time with no penalty, and their right to not answer any question they did not want.

All participants received a grocery voucher of 10.00 USD for their participation. As data collection and analysis was done iteratively, when saturation [40]—the point at which all concepts and categories were fully understood—was reached, recruitment was discontinued.

### Data collection and management

A thematic guide was used to gain insights about the participants’ views on malaria disease and on health research. Data collection was led in English by a male social scientist (GMP) with experience in qualitative research in sub-Saharan Africa, and aided by a local trained female co-interviewer who used ‘colloquia’ (Liberian English). No one else was present during data collection, which was done in a private office at the SJCH. Most participants, especially from KI2 and KI3, preferred to use ‘colloquia’ when answering the questions posed. Neither the social scientist nor the co-interviewer had any clinical or contractual relationship with any of the interviewees.

IDIs and FGDs were taped and were an average of 53 and 72 min in length, respectively. All recordings were transcribed verbatim in a password-protected computer. Transcriptions were cross-checked against the recordings. If there were inconsistencies, the transcripts were amended. All personal identifiers were removed from the transcriptions. Consent forms, recordings and transcriptions received a Unique Identification Number to enable linkage of documents. The transcripts were uploaded into Dedoose software (^®^SocioCultural Research Consultants, Manhattan Beach, CA). After data coding and analysis, all recordings were deleted to further protect participants’ confidentiality.

### Analysis

All transcripts were coded contemporaneously with data collection to ensure that all core concepts were addressed with the participants. No themes and codes were pre-defined. At first, data were line-by-line hand-coded using gerunds and making use of participants’ own words [40]. Once a final coding framework was defined during the first interviews, this framework was used to code the rest of the transcripts.

A feminist interpretation of grounded theory was used [41–43]. This interpretation involves that women participants are considered ‘co-generators’ of theory in cooperation with the social scientist. The social scientist is expected to practice reflexivity throughout her/his interactions with the participants, and to be sensitive towards issues of oppression and marginality. This approach prioritizes that research findings are useful for social change and to improve women’s health.

Different measures were used to guarantee the trustworthiness of this study. Participants’ answers from the IDIs were triangulated with their answers from the FGDs. During data collection, the social scientist kept a memo journal to reflect on the impact of his interaction with the women participants. Thoughtful care was put to map and analyse deviant cases. Throughout analysis and reporting, women’s own words were used to define concepts and categories. In the Results section, participants’ perspectives are expressed in their own words using *‘italics’*. As majority of participants’ narratives were in ‘colloquia’, excerpts have been edited for grammar correction. Excerpts have been carefully chosen to ensure they represent the findings and that the deviant viewpoints are also represented. Additionally, peer-checks were done on the final analysis. This article has been prepared as per qualitative research reporting standards set in the COREQ checklist [44].

### Ethics approval

The study was approved by the University of Liberia-Pacific Institute of Research & Evaluation Institution Review Board (Monrovia) and by the Hospital Clinic Research Ethics Committee (Barcelona).

## Results

Seventeen pregnant women (hereafter referred to as *women*), 11 traditional community representatives (the *leaders*), and 10 SJCH workers (the *staff*) participated (Table 1). There were no refusals. 26 IDIs and three FGDs were conducted. Five *staff*, five *leaders* and five *women* separately partook in the three FGDs. Thirty of the 38 participants were female. The median age was 35. Twenty-nine were born in Montserrado County. Sixteen and 11 participants had graduated from secondary and university education respectively. Unless stated otherwise, viewpoints reported in this section were common across KI groups.

**Table 1:** Basic socio-demographics of key informants (KI)

	Recording#		Sex	Department	Age group	County of birth	Education
	IDI	FGD					
KI1: SJCH Medical, Administrative and Laboratory *staff*	IDI5	FGD1	F	Medical	31–40	Montserrado	University
	n.i		M	Administrative	51–60	Montserrado	University
	n.i		F	Medical	31–40	Montserrado	University
	n.i		F	Medical	21–30	Montserrado	University
	n.i		F	Medical	31–40	Montserrado	University
	IDI4	–	M	Laboratory	31–40	Montserrado	University
	IDI6	–	M	Medical	41–50	Grand Bassa	University
	IDI7	–	F	Medical	31–40	Montserrado	University
	IDI15	–	F	Medical	31–40	Nimba	College
	IDI16	–	F	Medical	51–60	Montserrado	College

	Recording#		Sex	Traditional role	Age group	County of birth	Education
	IDI	FGD					
KI2: Traditional *leaders* participants to the SJCH Community Advisory Board outreach activities	IDI1	FGD2	M	Youth leader	31–40	Montserrado	Secondary
	IDI10		F	Chairlady	41–50	Montserrado	None
	n.i		M	Council of Elders	41–50	Montserrado	University
	n.i		M	Council of Elders	51–60	Montserrado	Secondary
	n.i		F	Chairlady	31–40	Montserrado	University
	IDI2	n.i	F	Chairlady	51–60	Montserrado	Secondary
	IDI3	n.i	M	Chief	41–50	Montserrado	University
	IDI8	n.i	F	Chairlady	41–50	Montserrado	Secondary
	IDI9	n.i	F	Trad. Midwife	61–65	Montserrado	Vocational
	IDI11	n.i	M	Herbalist	51–60	Montserrado	Secondary
	IDI12	n.i	M	Council of Elders	51–60	Montserrado	College

	Recording#		Sex	Current occupation	Age group	County of birth	Education
	IDI	FGD					
KI3: pregnant *women* participants in malaria prevalence study	n.i	FGD3	F	Student	18–20	Montserrado	Secondary
	n.i		F	Student	31–40	Montserrado	None
	n.i		F	Student	21–30	Montserrado	Secondary
	n.i		F	Business	31–40	Margibi	Vocational
	n.i		F	Student	31–40	Sinou	College
	IDI13	n.i	F	Student	21–30	Montserrado	Secondary
	IDI14	n.i	F	Student	21–30	Rivercess	Secondary
	IDI17	n.i	F	Business	21–30	Montserrado	None
	IDI18	n.i	F	Student	21–30	Montserrado	Secondary
	IDI19	n.i	F	Business	21–30	Bong	Secondary
	IDI20	n.i	F	Business	21–30	Montserrado	Secondary
	IDI21	n.i	F	Student	21–30	Montserrado	Secondary
	IDI22	n.i	F	Student	21–30	Nimba	Secondary
	IDI23	n.i	F	Physician Assist.	21–30	Montserrado	College
	IDI24	n.i	F	Student	21–30	Grand Cape	Secondary
	IDI25	n.i	F	Business	21–30	Grand Bassa	Primary
	IDI26	n.i	F	Student	21–30	Montserrado	Secondary

*n.i* not interviewed: participants who only partook either in an IDI or in a FGD

### Understanding the utility of research

Educating the general population on the role of health research is a much needed investment. As explained by the *staff*, even healthcare professionals were not being trained in the fundaments of research at the university. The situation worsened among the low-income population. As one *leader* explained, education on research needs to target especially the people ‘*in the rural’* and those ‘*sleeping in the muddy areas’* in Monrovia.

When asked to express, in their own words, what research entitled, most described that its aim is finding *‘solutions’* to people’s problems. There was consensus that the main contribution of malaria research would be the development of more acceptable preventive tools. The *leaders* also expressed that research could contribute with data useful to motivate the women not to ‘*overlook’* malaria.

To exemplify the potential utility of malaria research, one *leader* recalled what the contribution of research during the Ebola epidemic was:

*In research you are going to find what medicine you can do to help people fight the disease that is coming. Take for example the Ebola. When it came to Liberia; nobody had any idea! Many people died. But later on they got an idea when they did that research.* (IDI 2)

Many participants, including *staff*, mistook research for public health and healthcare provision. As argued by *staff* and *leaders*, lack of clear understanding on how research differs from other interventions could make pregnant women expect preventive, diagnostic or therapeutic benefits. If these expectations were not met, they would be disappointed. Thus, to build trust in the population, it would be crucial to promote education on the long-term societal benefits of health research in general and of malaria research in particular.

### Capitalizing on past experiences to inform malaria research

Participants’ knowledge on past research experiences was explored. Some participants mentioned public health interventions instead: humanitarian aid to war refugees, polio and measles vaccination, male circumcision campaigns, and community sanitation. A few participants reported that they were themselves engaged as surveyors in malaria awareness carried by *‘Youth Save’*, as community mobilizers for *‘Red Cross*’ during the Ebola crisis, or as health promoters for *‘Médecins Sans Frontières’* during a cholera outbreak.

Some of the methods witnessed in public health interventions were mentioned as ideas to plan future malaria research. For instance, as one *woman* described, field research staff could be selected *‘in a raffle’*, as allegedly done by organizations such as *‘Mercy Corps’.* One *leader* suggested that some of the communication strategies used during the war—such as calls for blood donation—could be used to motivate women to accept giving blood specimens during malaria research. The use of *‘drama’*, which was used during the post-war time, was also proposed to mobilize women:

*The UNMIL Mission in Liberia for peace*-*keeping. They had a peace message to give out. They used to have a comedian. He’d go, he’d perform, and you’d see people come. After performing, they [the UNMIL staff] would come and give the message.* (IDI 4)

The only health research mentioned was the *‘PREVAIL Ebola vaccine’* and the *‘Z*-*Mapp drug’* trials. Insufficient information to the population on these trials gave rise to fears about the safety of the experimental interventions. These fears were described as potential deterrents for pregnant women to engage in malaria research although only a few participants admitted sharing these fears. Three *staff* disclosed that they had worked in an Ebola Treatment Unit (ETU) where participants to the abovementioned trials were recruited. One of them said that she decided not to participate in PREVAIL after she witnessed how a colleague ‘*took the Ebola vaccine’* and ‘*got signs and symptoms of Ebola’*. One *staff* and one *woman* explained how they had heard that the *‘PREVAIL vaccine’* and the ‘*Z*-*Mapp drug’* were ‘*killing people’*.

*Doctor [Name of local physician] was one of those who took the vaccine and said: ‘As an example, I’m taking the vaccine, and it is not harmful’. But when [later] he died, many people started saying: ‘The doctor died. He took the vaccine, and that killed him’. It was not official. But people were discussing it in street corners.* (IDI 4)

The belief that people were given Ebola intentionally at the ETUs through *‘injections’* may lead some women to refuse the administration of experimental vaccines in the future. As one *leader* explained, during a recent polio vaccination, some mothers were reluctant to vaccinate their children because they suspected that unidentified foreigners were *‘bringing the Ebola vaccine to kill people!’* Some women may also refuse to give blood specimens because another common belief was that the Ebola virus was *‘man*-*made*’. Hence, as one *staff* expressed, some women may refuse to participate in malaria research in order to prevent their blood being used to ‘*fabricate other viruses again’*:

*I saw it on the internet. That Black American defending that Ebola was not a virus, that it was a chemical that they produced, and that the scientists were checking whether it could kill. And then the President Ellen Johnson Sirleaf and her colleague [Queen Sheba] agreed that they should come to try it in Liberia.* (IDI 22)

### Interacting with healthcare workers: barriers to overcome

Throughout all IDIs and FGDs, the participants reflected on how *‘rampant corruption’* and the interactions between healthcare workers and their patients could influence their own as well as other people’s attitudes towards malaria research conducted in healthcare institutions. Corruption was described as a problem that impregnated all aspects of public life in Liberia. ‘*Bribes*’ were described as commonplace in government-run facilities. One *woman* thought that this was the consequence of the government *‘taking long to pay staff salaries’.* Healthcare workers were also suspected to supply *‘drugstores’* nearby with anti-malarial drugs taken from the clinics. All *women* expressed that many pregnant women *‘feel discouraged’* when healthcare workers refuse to give them the prescribed drugs for malaria: *‘Little money! Any buddy you meet. They say: ‘Pay small thing’?’* In general, the participants were not optimistic about what women could do to ensure their right to healthcare access:

*The government is not able to fight the corruption because the corruption starts from up there before it comes down… So, how are we the citizens going to fight the corruption when the corruption is rampant within the government?* (IDI 22)

In addition to corruption, many Liberians may feel that they must accept anything prescribed to them by the health personnel. This widespread attitude of healthcare workers was identified as a potential deterrent for pregnant women to trust experimental treatments in future trials. As one *staff* explained, ‘*patients’ right’* to negotiate their pathway care was a foreign concept to the population:

*When I was not a medical practitioner, I used to think that if I had to go to the doctor to seek for medical care, and then the doctor suggests anything to me, if I refuse, he or she will not pay attention to me.* (IDI 4)

The fear to discover that the blood of participants might be *‘sold’* by research staff was sustained by awareness that doctors and nurses consented for blood to be traded in the clinics. Many participants described how some individuals made a living by selling their blood directly to the patients at the hospitals. Two *leaders* narrated their own experiences buying blood at two different hospitals in Monrovia. One *woman*, who disclosed in one FGD that she had to buy blood for each one of the three C-sections that she had, defended this practice because the hospitals needed to recover the investment they made to keep the blood refrigerated.

*‘Nothing for nothing’* was a motto frequently mentioned. In the frame of research, this notion would be *‘running in people’s minds’* because, as one *woman* explained, many would be convinced that researchers would sell their blood and personal data. The majority of participants suggested that pregnant women could be compensated financially for their participation in research. However, offering money may have downsides. *‘Poverty’* could unduly influence women to accepting to become trial subjects. Two *staff* claimed that they knew research staff from *‘PREVAIL*’ and previous HIV surveys who complained that people tried to enrol several times in order to receive the retribution. As *staff* and *leaders* expressed, autonomy of women will be compromised:

*Money! You enticed me to do it, but it might not be in my mind, it might not be my will to do that.* (IDI 4)

In addition to poverty, many pregnant women faced difficulties to access healthcare. Liberia has a mixed public–private health system, characterized by a very sparse network of health facilities. The population used two currencies: United States Dollars and Liberian Dollars. Fluctuations in the value of the Liberian Dollar affected access to clinic-based care. Hence, many participants claimed that, to ensure research findings could translate in improved prevention and therapies for pregnant women, it was key to ensure free access to healthcare in government facilities so that people could *‘save their money’* rather than spending it in *‘little somethings’* and *‘drugstores’*.

As a result of their experiences at the clinics, many pregnant women distrust healthcare workers, dislike the healthcare system, resort to *‘country medicine’* against their will, and self-initiate treatment with antipyretics and anti-malarial drugs that are easily available at *‘chemist shops’* and ‘*black dealers’* (street vendors). As expressed by the participants, research could be useful to encourage women to seek biomedical healthcare and to demand their rights as patients. Malaria research could be helpful not only to develop new prevention and therapeutic means but also to identify all healthcare access problems that pregnant women face in Liberia. However, as discussed by one *leader*, if the healthcare system did not discontinue its pay-per-service system, the community would miss the opportunity to benefit from improved malaria control interventions.

### Mobilizing the communities to engage in malaria research

Counting on general education alone would not be sufficient to promote the participation of pregnant women in research. *‘Intensive positive research awareness campaigns*’, as one *staff* put it, should be thoroughly planned in cooperation with the community leaders, and promptly put in place ahead of the conduct of malaria research. *‘Announcements’* to the population may be done in market places, taxi ranks, churches, video clubs, and football fields. Organizing ‘*forums’* or *‘palava hut discussions’* at schools, city halls, clinics, or at ‘*the junction of the road’* were proposed as preferred approaches to reach the communities. Going ‘*house by house’*, what two *leaders* termed ‘*Jehovah witness campaign’* was also valued as highly effective. Irrespective of the approach, any community-based mobilization must emphasize what the purpose of research is and what its potential individual and community ‘*disadvantages’* are.

Community mobilization for research should integrate health promotion activities for pregnant women explaining the aetiology of malaria as well as the most effective prevention, diagnostic and curative interventions. As rumours on experimental drugs and vaccines tested in previous trials might have originated fears and suspicions, two *staff* expressed that emphasis must be put in explaining to the communities that the only mean to develop better prevention and treatment tools is via the conduct of clinical trials:

*In the past there were no drugs for malaria. So people went and studied about the disease and how to prevent it. So, they did trials, they took samples and then they tested [a drug in] animals and then they tested it on humans. Over and over. And then they found out that it was good to treat malaria.* (IDI 7)

In agreement with *women’* and *staff*’s suggestions, the *leaders* partaking in a FGD described the methodological approach of this study as an example of what researchers were expected to do: leave their hospitals or academic institutions; reach the communities and their traditional representatives; provide training on the fundaments of research; and collaboratively mobilize pregnant women.

*You are the first person I see! I am fifty eight now. I live here. I was born here. I never saw people. So, if you tell me that we’ve got Liberian researchers… maybe they are doing research in their offices or in their bedrooms!* (IDI 11)

Chiefs and chairladies were described as *‘role models’* for the women that can authorize research, vaccination campaigns, and health promotion. As one female *leader* explained, if she were not engaged in the research, she would instruct the women *‘not to talk to the researchers’*.

*‘Sincere’* information on the research purpose and on the planned measures to safeguard pregnant women’s confidentiality must be given to leaders, women and their partners, for them to make their own informed decision on whether to participate or not. Researchers will be expected to disclose the potential side effects of the experimental interventions and on the study tests. Importantly, researchers will need to reassure women that they ‘*will not be harmed’*. If convinced about the safety of the study, as one *leader* expressed, some community representatives would even participate or allow their relatives to participate:

*In Africa people build confidence in the leader. And the leader must lead. If the community people don’t see me take the first step… So, to set an example, I would first allow my children to participate. And then the community would say: ‘Oh! If you, of all people, can do it, then we will do it’.* (IDI 3)

Many Liberians may mistrust researchers. Information on who the ‘*White researchers are’* will be expected. One *leader* proposed to describe researchers to women as people with ‘*humanitarian tendency’* who ‘*have love for life’.* As one chairlady expressed, she would tell the women that: *‘The white man is not here to kill you!’*

Endorsement of the research by their traditional representatives will be essential. All *women* in this study explained that they would not consider that the researchers are ‘*serious’* unless they had heard ‘*announcements’* from the leaders. Once researchers are introduced formally, to build rapport among women, they must be ‘*polite’, ‘honest’,* and *‘humble’*. And they must use *colloquia*. A common complaint about researchers was that they talked ‘*fast, fast’* to induce people into accepting to engage in research. The most common perception was that researchers ‘*become rich’* by developing treatments that they would never bring back to Liberia. As one *staff* complained, the Liberians are ‘*the sacrificial lamb’* whilst the researchers reap the benefits.

*They think you are picking the information to sell it down. Because some people think that many people who come on the field to educate people on HIV, malaria, at the end, they will say they will bring medicine, but they won’t come back.* (IDI 25)

### Understanding tradition to better plan research

The participants identified how, due to poorly designed communication on preventive measures during the Ebola outbreak, some Liberians were discouraged to adhere to the recommendations made by the health authorities. Many refused to believe that eating ‘*bush meat’* could lead to acquiring Ebola because the authorities’ messages did not consider the cooking habits of the most impoverished. Some other people believed that Ebola was not caused by a virus but by their ‘*forefathers’* who ‘*were vexed’*. According to the participants, the lesson learnt from the Ebola outbreak is that cultural practices and beliefs need be mapped and understood when planning research on malaria or other diseases.

Engaging women in malaria research will be challenging when they hold on to traditional beliefs on the causes of infectious diseases. Many Liberian women, even in urban areas, might think disease—including malaria—may be caused by *‘witchcraft’* and *‘evil water spirits’*. Some people also believed in offering *‘sacrifices’* to their *‘forefathers’* to put an end to their ailments:

*People that died 50, 60, 40* *years ago: we still believe that they live among us. So, if the farming season is bad, we make sacrifices. We consult them, ok? Then, somebody would interpret that since we had done sacrifices, things will be all right.* (FGD 2)

In this regard, research could be useful to help women disregard what they perceived as harmful beliefs that prevented them from seeking biomedical care for themselves and for their children. Women holding these beliefs may think that what affects them demands priority care from the *‘herbalists*’.

The younger generations in Liberia were confronting the rationale behind many traditional beliefs and were embracing *‘Western’* medicine. However, as one *leader* asked to other FGD members: *‘How can we get rid of the people’s belief? That is the question.’* Some perceived that health research could bring about change by helping people stop believing in ‘*witchcraft’* and ‘*forefathers’* as causes of disease and in *‘country medicine’* and ‘*sacrifices’* as methods to address them. Women would not feel offended if researchers, accompanied by leaders, advised against ‘*country medicine’* and *‘sacrifices’*. However, as most participants believed, advising women against the initiation ceremonies of the ‘*Sande secret society’* would be overly insensitive. Additionally, acknowledgement or suspicion that discussions on number of partners, abandonment of fathers of pregnancies, and illegal abortions might take place in the frame of research could make some women not accept to engage as study participants. One *staff* described these discussions as potentially ‘*embarrassing’*.

### Mapping fears, inconveniences and expectations

Some pregnant women may not agree to give blood for research purposes because they may fear being diagnosed—against their will—with a stigmatizing disease such as HIV. In addition, some women might also fear that research staff takes the occasion to *‘inject’* them *‘a disease’* or a harmful substance to either make them ‘*infertile’* or to ‘*impregnate them*’.

As some people may fear that blood specimens could be marketed or used for other purposes, researchers’ choice of methods for collecting and testing blood could also influence women’s consent to give blood. People would be more comfortable if *‘saliva’*-based rapid tests were used rather than ‘*venous blood’* tests. When no alternatives to blood-based tests existed, thorough face-to-face demonstration on the specimen collection procedures could be useful to allay women’s fears:

*You tell them: ‘We need to take blood from you to test you right now in your presence for malaria. This is the test. This is the syringe. This nurse will take your blood and test you straightaway.* (IDI 22)

The risk of social harm and of breaches in confidentiality was described as a potential deterrent for women targeted as participants that researchers should consider seriously. Many women could feel *‘shy’* to participate in interviews on malaria. Especially if women feared the interviews would be aired *‘on the radio or on the television’*, and if they feared the researchers would *‘judge their lifestyle’*:

*If you contracted the malaria because you were not using a treated mosquito net and the researcher asks you: ‘What do you think your problem is? Why do you come down consistently with malaria?’ You don’t want to tell the researcher: ‘I am not using a mosquito net’.* (IDI 3)

Community mobilization will be helpful to allay these fears. During mobilization, women and leaders who had previously participated in research could be invited as peer-mobilizers to reassure other women that no *‘side effects’* are associated to the experimental interventions. Some women would agree to participate if they see that *‘nothing happened’* to other women or if they see that the chiefs and chairladies consented for their own children to enrol.

### Framing gendered norms that could deter participation

Traditional gendered values and how these are used by community members to construct norms on how men and women should interact also need be considered in malaria research planning. Women were described as used to discussing sensitive topics with male midwives and nurses in Monrovia. However, in rural settings, as one *nurse* from Lofa County explained, women could be punished if seen talking to *‘strange’* men:

*All the women would be a distance away from the [male] strangers. You will never see women with your eyes. Why are the women chased away? That is traditional. If you, a man, touch any woman, they will beat that woman 50 lashes.* (FGD 1)

Men’s attitude to control women might be a barrier for pregnant women to engage in malaria research. Some *women* reported that they asked their partners to allow them to participate in the malaria prevalence study. One *woman* said she wanted to be sure she was not taking any risks. Other w*omen* in an FGD said that many asked for permission because of ‘*fear’* to their reactions.

For *staff*, *leaders* and *women*, Liberian men are *‘jealous’, ‘authoritarian’* and *‘believe in supremacy’* over women. Jealousy was described as an expected *‘manifestation of love’*, a demonstration that men are interested in their partners. Thus, some women asked them for permission *‘out of love’* because in Liberia women were expected to inform men about their engagement in any outdoor activity. In the opposite scenario of men being targeted as study subjects, no female participant in this study believed that any Liberian man would ever ask permission from his female partner.

The appropriateness of male research staff visiting women participants in their houses, in the scenario of a household-based malaria survey, was discussed. One *leader* elaborated that women would not have any problem but that their *‘boyfriends’* could create a *‘conflict’*. Some partners may also disagree with women being interviewed by a *‘white man’*. As one male *leader* explained, partners will feel that women, if visited by *‘young’* or *‘white’* men, they *‘may find another lover’*. To prevent conflicts, the use of community *‘elder men’* as *‘surveyors’* was suggested.

## Discussion

Government and academia-led science education, engagement of community leaders in research planning, and transparent information on the benefits that researchers and research institutions accrue from research could be useful strategies to improve pregnant women’s acceptance to participate in malaria research in Liberia. However, even if these strategies were adopted, a myriad of sociocultural factors, traditional beliefs and gendered norms could become deterrents for women’s actual engagement. If left unaddressed, these deterrents could easily hamper the conduct of research and hinder the transfer of improved prevention and care to the populations most at-risk of malaria.

The same factors that deter uptake of malaria prevention and care for pregnant women intertwine with community members’ views on the utility of malaria research. The participants’ arguments emphasize the need to address inequity gaps in the healthcare system to ensure that the most vulnerable benefit from the translation of malaria research findings into public health and healthcare provision. Inequity gaps in the healthcare system; low awareness of patients’ rights to negotiate care; lack of platforms to denounce violation of patients’ rights; paternalism from healthcare workers; and mistrust of healthcare workers were identified as problems that need be urgently addressed. On the other hand, participants’ justification of some current practices (i.e. payment for blood to help institutions recover their expenditure in equipment) may reflect the populations’ beliefs on how healthcare systems must be financed. Should this be the case, acquiescence of the population to palliate some of these inequity gaps might not be easily gained.

Suspicions, fears and misconceptions must be considered when planning malaria research in Liberia. Based on participants’ description of the *‘rumours’* that circulated during the Ebola epidemic, future research should include approaches that clearly explain the purpose of collecting bodily specimens, the procedures to collect them, and their destination not only to the participants but also to the communities involved. ‘Rumour surveillance’ strategies, such as the one implemented by the World Health Organization in 2004 during the avian influenza H5N1 outbreak [45], could also be useful for mapping doubts and concerns and providing a timely dissemination of accurate information for future health research studies.

Most of the fears and misconceptions described have been reported in other settings. For instance, fear to give blood that could be *‘sold’*, used to *‘fabricate’* other viruses, or tested for stigmatizing diseases has also been reported in Ghana [46] and in The Gambia [39]. Nevertheless, it must be noted that Liberia has not experienced as much malaria research as other West African countries. Current fears and myths gained momentum, according to the participants’ narratives, during the conduct of the *‘PREVAIL’* (ClinicalTrials.gov Id: NCT02344407) and *‘Z*-*Mapp’* (ClinicalTrials.gov Id: NCT02344407) trials [23, 27, 28]. These were two of the five trials initiated during the Ebola epidemics in Liberia [27]. Some of the beliefs and rumours reported in this article were already identified and addressed by the PREVAIL investigators at the very outset of the Ebola vaccine trial in Monrovia [23]. In spite of a social mobilization strategy put in place by the PREVAIL investigators [23], those fears and myths persist today. Irrespective of the alleged association of these *‘rumours’* with the Ebola vaccine trials, their persistence might also be explained by the population’s mistrust of health care workers and authorities, and might reflect broader concerns on current political and socio-economical issues. As issues pertaining to inequity and trust are not to be solved in the short-term, future research should not underestimate the possibility that these fears and *‘rumours’* may influence the acceptance of malaria research by pregnant women.

Some of the solutions proposed by the participants to improve people’s perception of research and to encourage participation have also been reported in other studies, such as the delegation of the explanation of the experimental intervention to local leaders and active mobilization through volunteer peer-education [47]. Interestingly, whilst previous studies reported on such approaches as effective solutions based on their actual research findings, in this study, the participants made these recommendations drawing upon the public health interventions they had witnessed during the war, post-war and the Ebola crisis. Thus, the possibility to translate site-specific acceptable and culturally-congruent practices from public health to malaria research should not be belittled.

This study has important implications for future research. The expressed possibility of undue inducement into research by means of financial compensation, reprisals from healthcare workers, and therapeutic misconceptions accentuate the vulnerability of pregnant women in this research context [34, 37, 48]. The possibility that some men may believe that women do not have the right to consent to participate in research on their own [37], a worrying consideration pointed out by the participants, also needs be tackled. To prevent these hazards, risk–benefit analyses must be performed at the outset of the research. Participatory approaches to research (PAR) in which the targeted communities and their traditional representatives are engaged, are key to put in place mitigation measures. As an example, during the Ebola outbreak, a research using PAR methodologies was conducted in New Kru Town, a low-income neighbourhood in Monrovia, to identify deterrents for pregnant women to seek for labour and delivery care in their government-run maternity referral hospital [49]. That research, which also engaged healthcare workers, community representatives and pregnant women, proved how PAR was a feasible qualitative approach to improve community members’ trust of healthcare workers and also to collaboratively plan strategies to promote healthcare utilization by pregnant women [49].

All potential barriers to malaria research conduct in Liberia are historically-shaped and relate to a wide range of factors. Looking exclusively at the impact of the Ebola epidemics in populations’ attitudes would be an erroneous approach. Fortunately, the participants of this study were able to discuss the different interview topics in the light of political, socio-economical and cultural nuances that transcended the Ebola epidemics. The aim of this study was to provide an accurate ‘picture’ of the current perceptions that may promote or hinder the pregnant women’s participation in malaria research, and not to interpret in depth how the recent history of Liberia is interwoven with this study’s findings. However, further socio-historical research could be done to analyse how the US-promoted neoliberal capitalism during the cold war times [50, 51]; the endemic corruption during the post-war mandate of President Ellen Johnson Sirleaf [52]; and the implementation of clinical trials during the Ebola outbreak [23, 28], might have shaped how the Liberians construct notions, meaning and values with regards to health, disease, healthcare and research.

The strength of this study was the use of the feminist grounded theory to identify areas of oppression and vulnerability for women [41–43]. Some of the issues of oppression identified by the participants, such as the need to extend research education to women’s partners and the women’s fears of being judged by healthcare workers for their uptake of malaria prevention, must be taken into account when planning future malaria research.

A limitation of this study was that a more comprehensive description on the deterrents to engage in malaria research could have been obtained if individuals with no history of engagement in research had been invited. Thus, the possibility that social desirability might have guided the participants’ narratives cannot be disregarded. Recall bias might also have also influenced the participants’ narratives of past malaria care-seeking and of exposure to trials during the Ebola outbreak. Another limitation was that no discussion was held on the participants’ views regarding the exclusion of pregnant women from research to avoid health risks to the foetus. Some participants may have been aware that the trials initiated during the Ebola epidemics excluded pregnant women as participants [27] and their opinions in that regard might have been very useful to this discussion.

## Conclusion

The participants of this study suggested implementing measures to provide evidence-based malaria and research education to the general population and to engage community leaders in research design, target population mobilization, and local dissemination activities. In agreement with the New Kru Town’s PAR study [49], researchers should consider investing time and financial resources to ensure community support in any strategy aiming at informing pregnant women of the purposes of the malaria research and in soliciting their participation. Benchmarking for acceptable practices used in previous public health campaigns as well as mapping, documenting and understanding implementation and communication errors from previous clinical trials during the Ebola outbreak might help allay fears and misconceptions and encourage pregnant women to engage in malaria research as participants whose rights to wellbeing, autonomy, confidentiality and safety are safeguarded. Importantly, if widespread disempowerment and inequity issues are not tackled, population’s lack of trust in the healthcare establishment and in researchers will hinder their acceptance of malaria research, and, importantly, might impede the translation of key novel findings into improved access and use of malaria prevention and therapeutics by the most vulnerable populations attending the Liberian healthcare facilities.
